# A 1.2 Billion Pixel Human-Labeled Dataset for Data-Driven Classification of Coastal Environments

**DOI:** 10.1038/s41597-023-01929-2

**Published:** 2023-01-20

**Authors:** Daniel Buscombe, Phillipe Wernette, Sharon Fitzpatrick, Jaycee Favela, Evan B. Goldstein, Nicholas M. Enwright

**Affiliations:** 1 Contractor, U.S. Geological Survey Pacific Coastal and Marine Science Center, Santa Cruz, CA USA; 2 U.S. Geological Survey Pacific Coastal and Marine Science Center, Santa Cruz, CA USA; 3 Department of Geography, Environment, and Sustainability, University of North Carolina at Greensboro, Greensboro, North Carolina USA; 4 U.S. Geological Survey Wetland and Aquatic Research Center, Lafayette, LA USA

**Keywords:** Physical oceanography, Databases

## Abstract

The world’s coastlines are spatially highly variable, coupled-human-natural systems that comprise a nested hierarchy of component landforms, ecosystems, and human interventions, each interacting over a range of space and time scales. Understanding and predicting coastline dynamics necessitates frequent observation from imaging sensors on remote sensing platforms. Machine Learning models that carry out supervised (i.e., human-guided) pixel-based classification, or image segmentation, have transformative applications in spatio-temporal mapping of dynamic environments, including transient coastal landforms, sediments, habitats, waterbodies, and water flows. However, these models require large and well-documented training and testing datasets consisting of labeled imagery. We describe “Coast Train,” a multi-labeler dataset of orthomosaic and satellite images of coastal environments and corresponding labels. These data include imagery that are diverse in space and time, and contain 1.2 billion labeled pixels, representing over 3.6 million hectares. We use a human-in-the-loop tool especially designed for rapid and reproducible Earth surface image segmentation. Our approach permits image labeling by multiple labelers, in turn enabling quantification of pixel-level agreement over individual and collections of images.

## Background & Summary

The availability of imagery from Earth observation platforms^1^ in coastal areas^2^ has enabled models of physical processes in the coastal zone to focus on coastal change measured in decades to centuries and tens to hundreds of kilometers^3^. Part of this shift is an increasing acceptance of the notion that large-scale coastal issues may only be addressed with large-scale measurements from aerial or space platforms, even if those measurements are less accurate than traditional ground-based survey measurements^4^ because of the relatively high temporal and spatial coverages of satellite-based measurements^2,5^. Remotely sensed photography has been used to monitor coastal ecosystems and hazards, such as hurricanes^6,7^, flooding^8,9^, and cliff erosion^10^, for almost a century. In some areas, aerial photos of the coast predate extensive modification of coastal morphology and ecosystems by humans.

Modeling coastal systems at large spatial and temporal scales requires methods to extract information from images. A traditional way to do this is through developing landcover maps. Modern landcover mapping efforts are designed to facilitate users bringing their own pixel classification and other image analysis algorithms to the data^11^, using petabyte-scale ‘analysis-ready’ data in cloud storage^1^, and carrying out accuracy and other quality assessments of the landcover maps informed by specialist knowledge (e.g., ecological or physical). This manual work is time consuming, hence the widespread interest in and adoption of automatic identification and mapping of natural or human-induced coastal change from geospatial imagery^12^. Coastal scientists are largely concerned with mapping features at and near the intersection of land and water, and with the visible expressions of water flows and seasonal growth patterns and other processes. While leveraging existing national- and international-scale landcover products is possible to a certain degree^5^, there is also a pressing need for labeled datasets for more and more specific land and water categories, relating to, for example, specific water and flow states, morphologies, sediments and surficial geologies^5,13^, habitats and vegetation types^14^, hydrodynamics, and coastal infrastructure^12^.

Time-series of coincident imagery can display transitions in habitats, morphologies and sediment distribution, as well as signatures of change or visible indicators of characteristics of a particular type of coastal landform or habitat, even without detailed measurements of elevation. For example, it is possible to use a segmented image time-series to estimate beach slope, and from that slope, a reasonable estimate of beach grain size might be inferred through the application of existing empirical models that relate grain size to slope^15^. Further, a time-series of segmented images, being classified at the pixel-level, is ideally suited to many coastal remote sensing tasks that require high-frequency information at event scales. Among numerous potential uses of segmented imagery, some examples include capturing the expansion, densification, displacement, or otherwise, of development at the coast^16^, disaster assessment such as inventory of development and land-uses in hazard zones^17^, geomorphic mapping^13,18^, mapping water for verification of flood-inundation models^19^ using imagery, and examining the effectiveness of coastal management practices such as interception of sediment transported by longshore drift by coastal structures on eroding coasts^20^, or beach nourishments, by mapping the spatiotemporal distributions of (at least) sand and water^21^. Some smaller publicly available datasets for segmentation of time-series of imagery of coastal zones already exist, for specific objectives involving highly specialized labels or specific imagery or coastal landform types^22–32^.

Our dataset consists of pixel-level discrete classification of a variety of publicly available geospatial imagery that are commonly used for coastal and other Earth surface processes research. The primary purpose is to provide coastal researchers a labeled dataset for training machine learning or other models to carry out pixel-based classification or image segmentation. The adoption and communication of a rigorous, reproducible, and therefore fully transparent accuracy assessment for coastal-specific labeled imagery is lacking, for example specific details about dataset creation, such as label error. One way to quantify labeling errors is to measure inter-rater-agreement in a multi-labeler context^33^, a practice adopted here. After all, any supervised image segmentation model and model outcomes that resulted from training on the Coast Train dataset would only be as good as the quality of that dataset^34^. Therefore, any quantifiable lack of agreement could be used as a conservative measure of irreducible error in model outputs.

## Methods

### Site and image selection

**Fig. 1:**
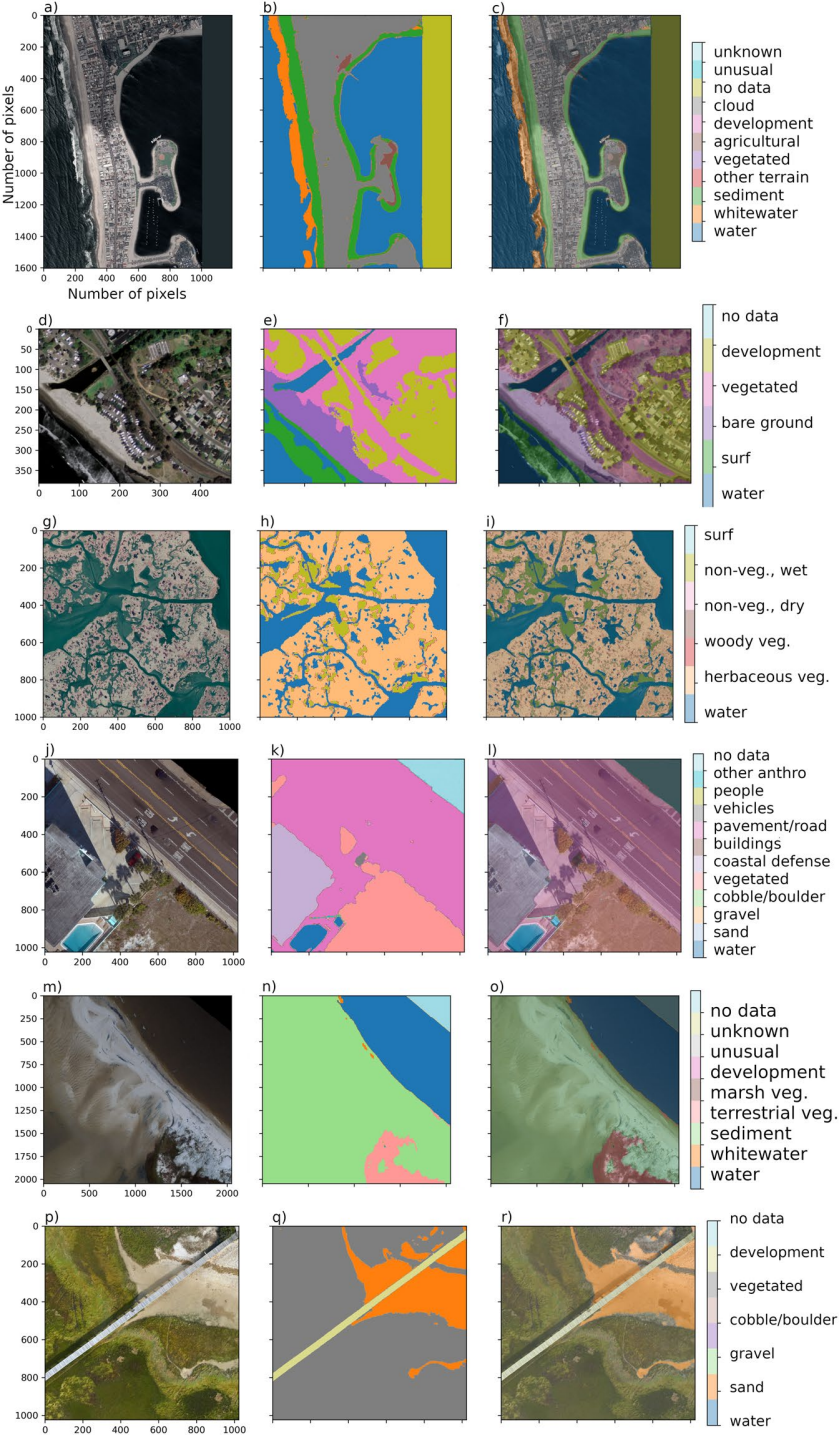
Rows (from left to right) depict one example image, corresponding label image, and image-label overlay, of each of the orthomosaic datasets. Columns show imagery from San Diego, California (**a**), Monterey Bay, California (**d**), Mississippi River Delta, Louisiana (**g**), Madeira Beach, Florida (**j**), Pelican Island, Alabama (**m**), and Sandwich Town Beach, Massachusetts (**p**).

**Fig. 2:**
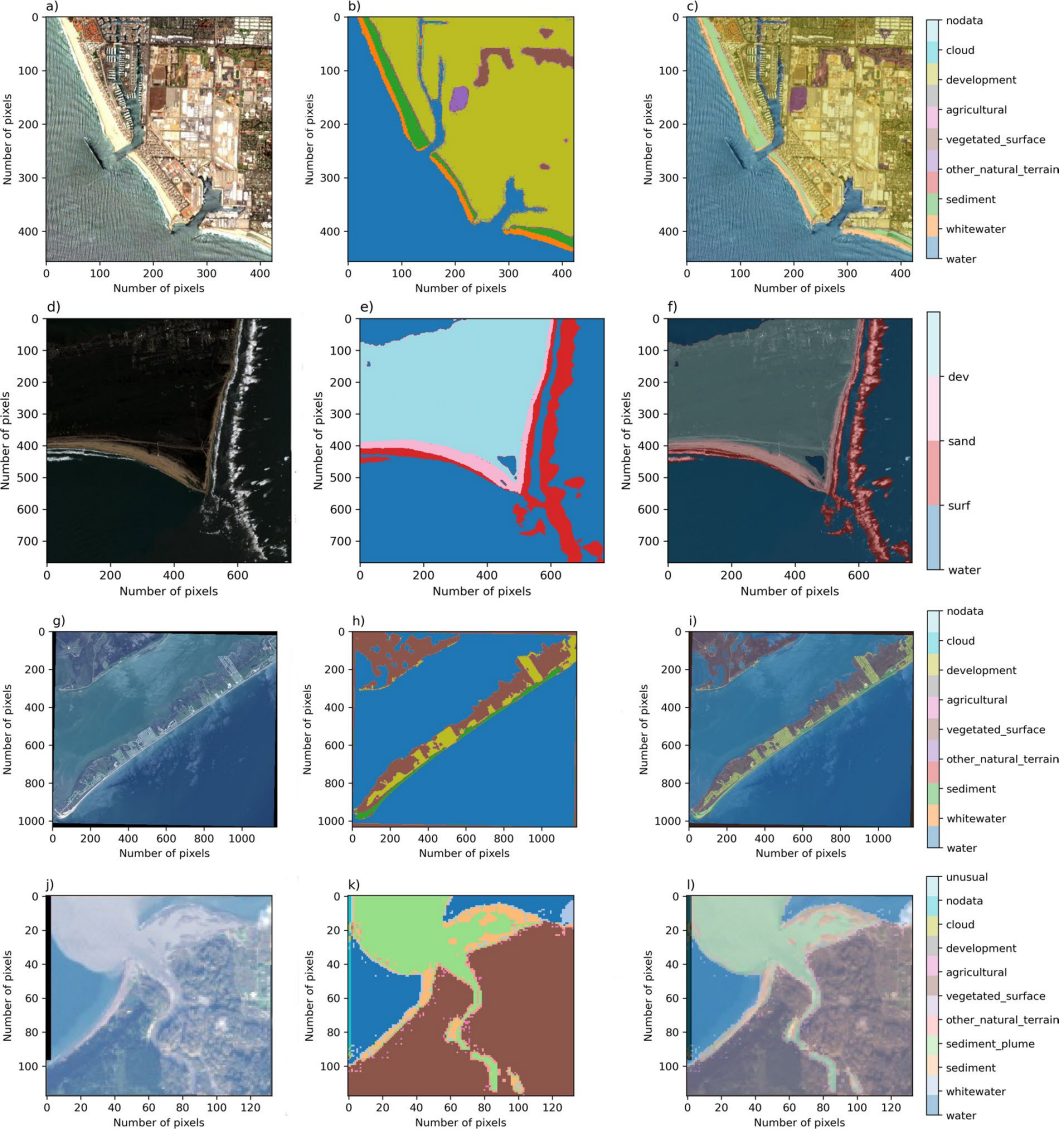
Rows (from left to right) depict one example image, corresponding label image, and image-label overlay, of each of the satellite image datasets. From top to bottom; Sentinel 2; Sentinel 2, 4 class; Landsat-8; and Landsat-8, Elwha. Columns show imagery from Ventura, California (**a**), Cape Hatteras, North Carolina (**d**), Galveston Island, Texas (**g**), Elwha River Delta, Washington (**j**).

The dataset^35^ consists of 10 data records, derived from 5 different imagery types, namely National Agricultural Imagery Program (NAIP) (aerial), Sentinel-2 (satellite), Landsat-8 (satellite), U.S. Geological Survey (USGS) Quadrangle (aerial), and Uncrewed Aerial System (UAS) -derived orthomosaic imagery. Each data record is characterized principally by the combination of image type and class set. Class sets are the lists of labels, or classes, used to segment the data. The study was confined to locations within the conterminous United States (CONUS), and locations related to various historical and present USGS research objectives within coastal hazards and ecosystems research were prioritized. Even within this scope, due to the large amount of imagery available and limited time to label in a multi-labeler context, which is more time-consuming than single-labeler contexts, we prioritized image sets according to geographic location, including multiple representative imagery from Pacific, Atlantic, Gulf, and Great Lakes coastlines. As described below, we included a set of relatively recently published sets of high-resolution orthomosaic imagery (Fig. 1) created from aerial imagery collected from following a Structure-from-Motion workflow^36^ in addition to geospatial satellite imagery data available throughout CONUS (Fig. 2). The orthomosaics are locationally specific data collectively represent muddy, sandy, and mixed-sand-gravel beaches and barrier islands, in developed and undeveloped settings.

### Image retrieval and processing

Sentinel-2 (https://www.esa.int/Applications/Observing_the_Earth/Copernicus/Sentinel-2) imagery was collected over the period 2017–2020, and Landsat-8 (https://www.usgs.gov/landsat-missions/landsat-8) imagery over the period 2014–2020. Sentinel-2 and NAIP (https://www.fsa.usda.gov/programs-and-services/aerial-photography/imagery-programs/napp-imagery/index) imagery was accessed using the parts of Google Earth Engine (GEE)^1^ Application Programming Interface (API) exposed by functionality encoded into the software program Geemap^37^, and Landsat-8 Operational Land Imager (OLI) imagery was accessed using the GEE API within the CoastSat program^38^. Only Tier 1 Top-of-Atmosphere (TOA) Landsat products (GEE collection “LANDSAT/LC08/C01/T1_RT_TOA”) and equivalent Level-1C Sentinel MSI (“COPERNICUS/S2”) products were used, which exhibit the most consistent quantization over time^26,39^. Imagery was orthorectified by the data provider, and no image registration was carried out. All Landsat imagery were pan-sharpened using a method^40^ based on principal components of the 15-m panchromatic band, resulting in 3-band imagery with 15-m pixel size. Visible-band 10-m Sentinel-2 imagery was used. Landsat-8 imagery was masked for clouds using the provided Quality Assessment band that includes an estimated per-pixel cloud mask, whereas only cloudless Sentinel-2 imagery (assessed visually) is used because a per-pixel cloud mask is not available. Though spectral indices that contain the Short Wave Infrared (SWIR) band such as the Modified Normalized Difference Water Index (MNDWI) have been shown to facilitate more reliable automated classification of water bodies in coastal regions^25,38^, only pansharpened visible-spectrum (blue, green, and red bands) imagery were labeled. Additional coincident spectral bands are available for each satellite scene at the same spatial resolution, for example near-infrared and shortwave-infrared bands; the labels created using the visible-spectrum imagery would apply to those additional bands.

Cloudless 1-m NAIP orthomosaic imagery was collected at various times in summer between 2010 and 2018. There are 493 images depicting 366 unique locations. USGS quadrangle imagery (https://www.usgs.gov/centers/eros/science/usgs-eros-archive-aerial-photography-digital-orthophoto-quadrangle-doqs) depict mud-dominated delta and wetland environments of the Mississippi River Delta in Louisiana, collected in summer 2008 and 2012. To represent sand-dominated Gulf Coast environments, we include 5-cm orthomosaic imagery created from low altitude (<100 meters above ground level) nadir imagery of portions of Dauphin Island (Little Dauphin and Pelican Islands), Alabama^41^, and Madeira Beach, Florida^42^. Mixed sand-gravel beaches are represented in our dataset using 5-cm orthomosaic imagery created from low altitude imagery collected between 2016 and 2018 at Town Neck Beach in Sandwich, Massachusetts^43^. All 5-cm orthomosaic imagery was downloaded in GeoTiff format, tiled into smaller pieces of either 1024 × 1024 × 3 or 2048 × 2048 × 3 pixels depending on the dataset using the Geospatial Data Abstraction Library software^40^ (https://gdal.org) and converted to jpeg format prior to use with Doodler (https://github.com/Doodleverse/dash_doodler), our labeling tool that creates dense (i.e., per pixel) labels from sparse labels called doodles^44,45^, further described in the ‘Image Labeling’ section below. All imagery data are provided in unsigned eight-bit format.

### Class set selection

We convened an invited panel consisting of seven experts on topics concerning coastal imagery, Land-Use Land-cover (LULC) and Machine Learning (ML) and met virtually for two hours to discuss the project and to determine a set of class labels for use with the various image sources and coastal geographies. During this meeting the various strategies we later adopted were proposed and discussed. The most important decision was to create a custom class set or two per image type, using a short list of simple (broad/elemental) classes, with the option to build complexity later (i.e., a hierarchical labeling approach). Addressing coastal challenges using ML and image libraries requires that the classes match the features that can be readily distinguished in the images. Because some features are distinguishable in UAV imagery that are not distinguishable in aerial or satellite imagery, it is reasonable to use a different class list for the different image sources. Coast Train represents this scalable image and label library and includes a range of sources, from very high-resolution UAV images to spatially coarse but higher spectral resolution satellite images. In this way, the image and label library can be more readily utilized to address a range of coastal issues compared to existing land cover data derived solely from coarser satellite imagery.

During the expert panel discussion, it was also decided that water and whitewater would ideally always be included as categories as all the imagery depicted shoreline environments. We also defined class sets that could be combined into meaningful superclasses. In ontology, a superclass is a broad class name for a collection of subclasses. In this dataset there are seven superclass labels, and between four and 12 class labels. During the expert panel meeting it was further decided that we would only label what we are confident about, to maximize true positives in the training data by including relatively simple and unambiguous classes and a probability sink (unknown/uncertain) class. For some image sets we also adopted a suggestion of using an ‘unusual’ class to describe things that are not in the class set but occasionally appear in the scene. Finally, the utility of image sets with overlapping geographies was also posited, foreseeing the utility of linking relatively high- and low-resolution imagery. Linking class lists to the input image resolution (spatial and spectral) is important as some features like beach umbrellas, construction equipment, and woody debris are resolvable in higher resolution images but may become aggregated with the surrounding landscape in coarser resolution images. For these reasons, each image set was labeled using its own class list (Table 1).

**Table 1:** Dataset summary.

Name	Publisher	Number of images	Pixel size (m)	Number of unique scene locations	Number of labeled pixels (million)	Number of classified pixels (million)	Classified ground area (hectares)	Number of classes
NAIP-11 class	USDA	493	1	366	63.720	380.227	38022.723	11
Quadrangles	USGS	44	6.83	25	2.892	44.122	4412.208	8
Sentinel 2–11 class	Copernicus	340	10	99	28.25	67.088	670878.850	11
Sentinel2-4 class	Copernicus	103	10	2	9.863	44.205	442045.440	4
Landsat-8	USGS	350	15	8	21.572	108.596	2443414.838	11
Landsat-8 (Elwha River)	USGS	50	15	1	0.771	1.133	25483.500	12
Madeira Beach	USGS	26	0.05	26	4.371	27.263	6.816	12
Dauphin Island	USGS	42	0.05	42	15.984	174.916	43.729	9
Sandwich Beach	USGS	289	0.05	289	17.695	286.653	71.531	8
NAIP-6 class	USDA	115	1	79	4.037	58.41	5841.465	6
Totals:	n/a	1852	n/a	937	169.115	1192.617	3630221.100	n/a

### Image labeling

We achieved a fully reproducible workflow by using a semi-interactive ML program called ‘Doodler’^44,45^ that uses sparse labels contributed by human labelers to estimate classes for all pixels. Its use in the Coast Train project is designed in such a way that each label image may be reconstructed using the sparse labels provided by a human labeler, and further, those labels might be repurposed using a different algorithm, if necessary. This idea ensures reproducibility and is articulated further in a companion paper^44^ that is based on a similar dataset^45^ that complements the one described here but is much smaller and spatially and temporally less extensive. The level of reported detail surrounding new human-labeled datasets is often poor, including the minutiae of decisions and other details that might impact the subsequent use of the data^34^, so below we describe how the labeling team interacted over the tasks.

### Label quality assurance

It is common to divide the work of labeling data among a group of people, which allows the labeling to be carried out in a shorter time period. However, group labeling in this way does not allow for quality assurance such as flagging outliers and measuring inter-labeler agreement^44^. We adopted a hybrid approach where some datasets were labeled by a single individual, for time efficiency, but also several datasets contained many images that were labeled by two or more individuals, ensuring sets of labels that could be compared quantitatively^44^, a procedure that is described below.

The labeling task required an ability to recognize coastal landscapes and processes and, to a lesser degree, knowledge and experience with the Doodler program and the rudimentary elements of the ML behind it. The group of labelers had diverse backgrounds and career stages. The labeling team comprised two early career scientists who had limited prior knowledge of geosciences, and another who had a geoscience background with limited experience of data science and software. These individuals had never participated in data labeling tasks before. Other labelers had a wide range of experience with data labeling tasks involving geoscientific imagery. To ensure those respective backgrounds and experiences introduced minimal bias, and to otherwise ensure consistency among labeling styles and maintain high standards in the outputs, we adopted a practice of training and frequent communication.

First, the labeling team took training, during which the Doodler program^44^ was explained, demonstrated, and questions over its usage answered. Labelers were trained on how to load images, modify class lists, and were provided examples detailing how to add/edit/remove doodles. The adjustable post-processing and classifier settings available were explained; however, users were encouraged to edit doodles before altering default settings. This approach was because it is generally faster and sufficient to change pre-existing doodles than modify settings^44^. The human-in-the-loop aspect of the Doodler program places emphasis on humans aiding machine learning labeling of images; instead of only the human or the machine labeling each of the classes in the image, they work together for quicker and more consistent/objective labeling. This approach places emphasis on gaining user experience with the program. Therefore, each labeler spent time practicing with the program before being assigned Coast Train imagery to label. There is a short learning curve for each individual as they develop their own labeling style and a relationship with the program that guides how much labeling is required, how to edit, and re-segment the image until they are satisfied with the results. Once such a relationship is established, labeling becomes a quick process and valuable labeled images are produced.

Thereafter, messaging and videoconferencing were used to establish criteria for sufficient labeling, receiving advice and consensus over images and portions of images that were hard to identify, and checking for consistency among different labelers, to ensure the same strategy among different labelers was used to produce similar results. The labeling team was in hourly and daily communication via an instant messaging service, as well as during weekly meetings via videoconferencing. During weekly meetings, progress and challenges were discussed, and new tasks assigned. During these meetings, labelers were given a preview of the new dataset they were to label and warned of challenges they would need to overcome to successfully label the dataset. The challenges discussed typically related to (a) identifying features in relatively low-resolution satellite imagery and (b) the presence of new classes not previously encountered by labelers. Following the meetings, the labelers independently reviewed and labeled each image using the classes given during the meeting. In addition, we collectively carried out the analyses presented by a companion paper^44^, after which we were satisfied that varying interpretation and labeling styles impacted resulting labels minimally. We present similar label agreement statistics later that confirm this initial observation on the current dataset.

## Data Records

There are 10 Coast Train data records^35^ (Table 1), including six orthomosaic-derived datasets and four satellite-derived datasets. Each dataset is associated with a specific image type and class set. Among the class sets, horizontal spatial resolutions range between 0.05 m and 1 m for orthomosaics, and either 10 m or 15 m for satellite imagery. All image sources are publicly available. Orthomosaic imagery (Fig. 1) is included to represent specific coastal environments at 5-cm pixel resolution. NAIP (1 m), Quadrangle (~6 m), Sentinel-2 (10 m), and Landsat-8 (15 m) imagery collectively represent continental-scale diversity in coastal environments (Fig. 2) at a range of pixel resolutions.

**Fig. 3:**
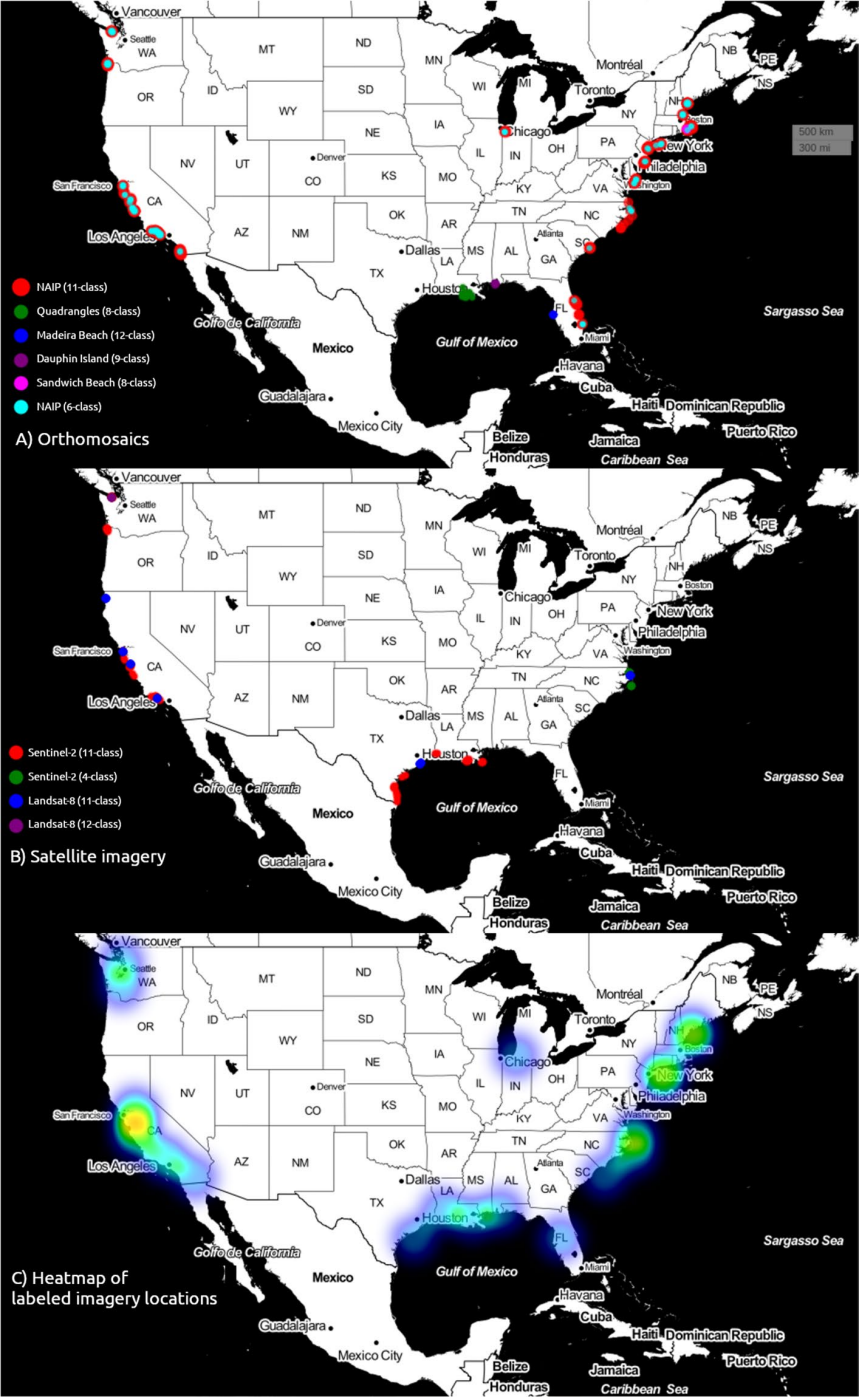
Geographical distribution of (**A**) orthomosaic and (**B**) satellite imagery, and (**C**) the ‘heatmap’ of image locations, or the number of images in spatial bins.

**Fig. 4:**
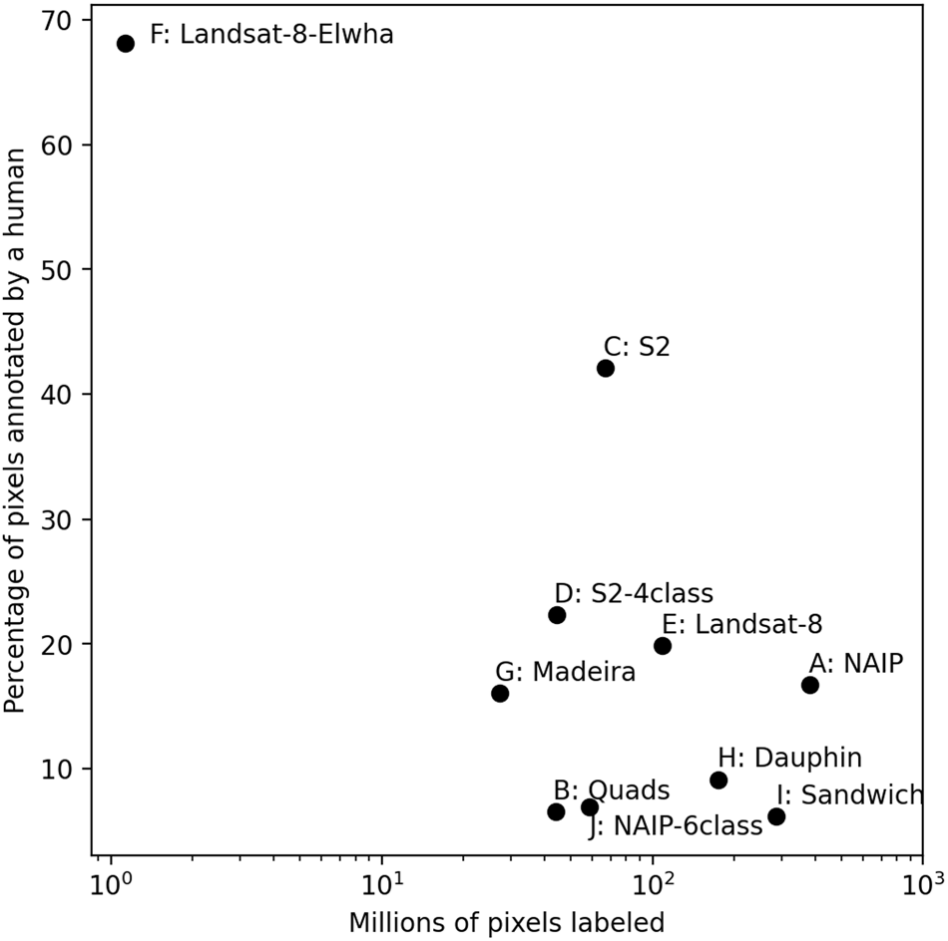
The size of the individual datasets, expressed as millions of total pixels labeled, computed as the product of the two horizontal label image dimensions, summed over all labeled images in each set. Percentage of pixels labeled by a human is computed as the product of the two horizontal label image dimensions and the proportion of the image labeled using the labeling program ‘Doodler,’ summed over all labeled images in each set.

The number of class labels varies between four and 12. The dataset consists of 1852 individual images, comprising 1.196 billion pixels, and representing a total of 3.63 million hectares of Earth’s surface. Most image sets are composed of time-series from specific sites, ranging between two and 202 individual locations. Sites were manually selected according to U.S. Geological Survey mission objectives, and to provide a large range of different coastal environments and locations in all regions of the United States. Other imagery covers an area at one specific time. Collectively, the data records have been chosen to represent a wide variety of coastal environments, collectively spanning the geographic range 26 to 48 degrees N in latitude, and 69 to 123 degrees W in longitude (Fig. 3). The labelers directly labeled 169 million pixels (about 14%); the algorithms in Doodler segmented the remainder (Table 1, Fig. 4). Each labeler performed on-the-fly quality assurance through diligent usage of the labeling tool.

Labels are reproducible; images and their corresponding label images are provided in a data archive per image file in compressed numpy^46^ npz format file format (https://numpy.org/doc/stable/reference/generated/numpy.savez_compressed.html, containing variables described in Table 2) that also contains all the file variables necessary to reconstruct the labels. Packages are available in every popular programming language to read numpy arrays. We provide codes to extract all images and labels and other information using utility scripts packaged with the Doodler program. It is possible to use Doodler to reconstruct all the labeled imagery from the original sparse labels (or ‘doodles’) that are recorded to file. Metadata files for each data record (Table 3) describe spatial footprint, coordinate reference system and many other details for each image and corresponding label image and are provided as tables in csv format, detailing one image per row.

**Table 2:** npz format file variables.

Variable	Description
‘image’	Image used by the Doodler program. This is the first 3 bands of ‘orig_image’
‘orig_image’	Original 8-bit unsigned integer image read by the Doodler program, that may contain 4 bands.
‘label’	One-hot-encoded label image (2D raster) in 8-bit unsigned integer. Each integer encodes a class label, incrementing through ‘classes’ starting at zero. Refer to^40^ for an explanation of and rationale for storing labels in one-hot-encoded format.
‘color_label’	8-bit unsigned integer 3D (RGB) version of ‘label’ colorized according to a discrete colormap
‘color_doodles’	8-bit unsigned integer 3D (RGB) raster of doodles colorized according to a discrete colormap
‘doodles’	8-bit unsigned integer 2D raster of doodles. It is possible to use Doodler utilities to reconstruct ‘label’ from ‘doodles’ and values listed in ‘settings’
‘settings’	List of settings used internally by the program, including the final values of the hyperparameters that may have been modified by the labeler
‘classes’	List of strings, each string a class name
0-prefix	The variables ‘label’, ‘doodles’, and ‘color_doodles’ may have one or several prefix zeros, the number of which indicate the order of the previous trial. Variables without a zero prefix are always the final versions.

**Table 3:** csv format file variables.

Field(s)	Description
‘annotation_image_filename’	npz format file containing the label data archive
‘classes_array ‘	names of possible classes in this dataset
‘classes_integer‘	one integer per element in ‘classes_array’
‘classes_present_integer’	one integer per element in ‘classes_present_array’
‘classes_present_array’	names of possible classes in this image
‘pen_width’	final width in pixels of pen used to doodle
‘CRF_theta’, ‘CRF_mu’, ‘CRF_downsample_factor’, ‘Classifier_downsample_factor’, ‘prob_of_unary_potential’, ‘num_of_scales’	internal classifier hyperparameters used by the Doodler program. Refer to^61^.
‘num_classes’	number of possible classes in this dataset
‘doodle_spatial_density’	proportion of the image doodled
‘acc_georef’	accuracy in meters of the specification of ‘XMin, XMax’ and ‘YMin, YMax’
‘epsg’	EPSG code of the projected coordinate system ‘CRS’
‘year, month, day’	time variables
‘hour, minute, second’	time variables
‘XMin, XMax’	minimum and maximum Easting of image footprint
‘YMin, YMax’	minimum and maximum Northing of image footprint
‘LonMin, LonMax’	minimum and maximum Longitude (WGS84) of image footprint
‘LatMin. LatMax’	minimum and maximum Latitude (WGS84) of image footprint
‘CRS’	the projected coordinate system description relating to ‘XMin, XMax’ and ‘YMin, YMax’
‘px_size_m’	horizontal size of pixel in meters
‘ImageHeightPx’, ‘ImageWidthPx’, ‘ImageBands’	Number of pixels in horizontal dimensions X and Y, and the number of bands (always 3)

## Technical Validation

The geographic distribution of labeled orthomosaic images (Fig. 3A), satellite images (Fig. 3B), and the number of images in spatial bins (Fig. 3C) show that the majority of coastal states within CONUS are represented. The final dataset contains numerous (but unequal) examples of coasts dominated by rocky cliffs, wetlands, saltmarshes, deltas, and beaches, including rural and urban locations, and low- and high-energy environments. The size of the individual datasets, expressed in terms of total pixels labeled, varies between ~1 and ~380 million (Fig. 4, Table 1). The percentage of pixels directly labeled by a human also varies considerably among individual datasets, from ~5 to ~70% (Fig. 4). The percentage of pixels labeled and total pixels labeled are negatively correlated; labelers tend to label a larger proportion of lower-resolution scenes. This is typically because more features are visible and must be identified in higher resolution imagery.

**Fig. 5:**
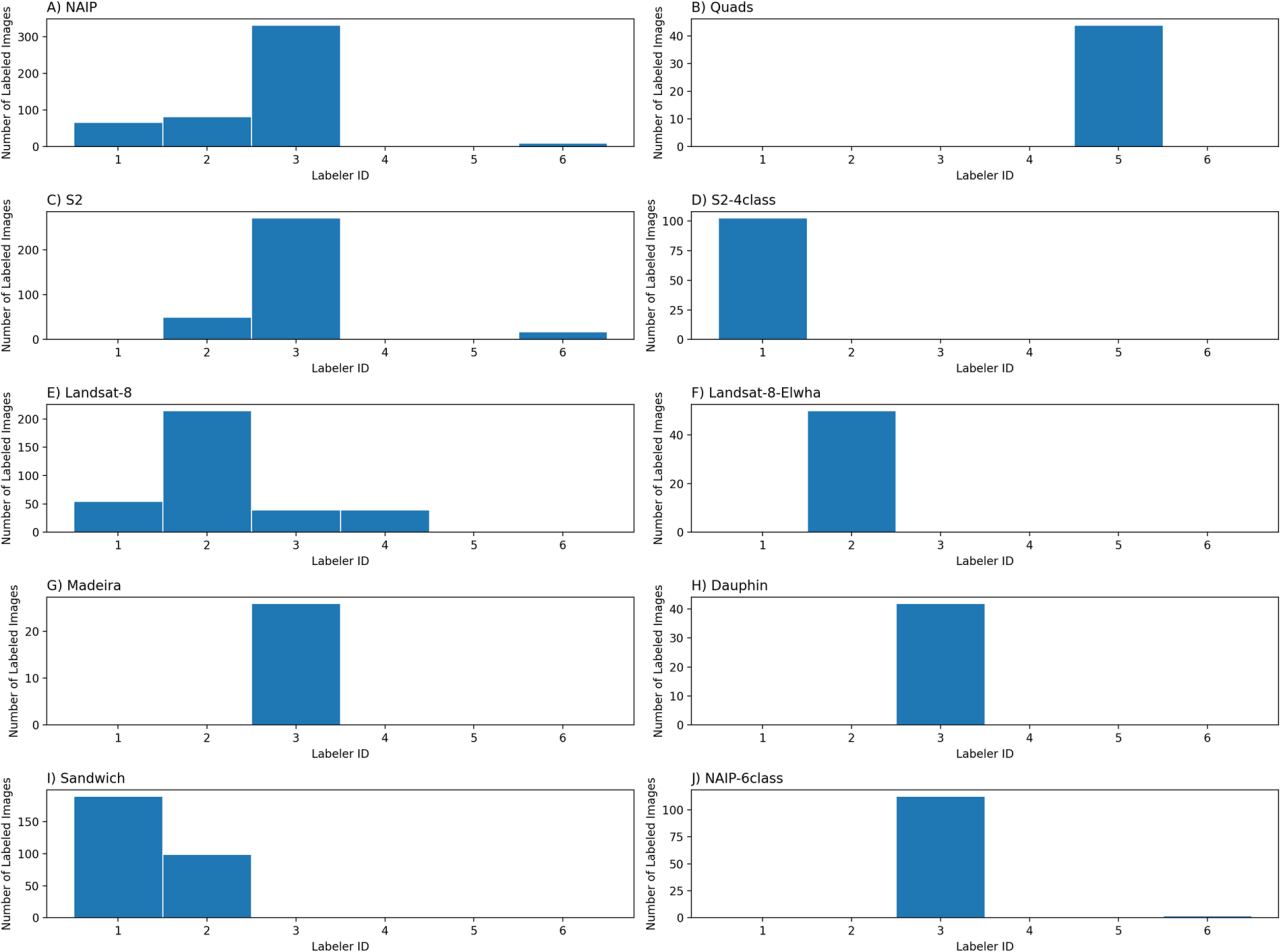
Frequency distribution of images labeled by unique labeler ID.

**Fig. 6:**
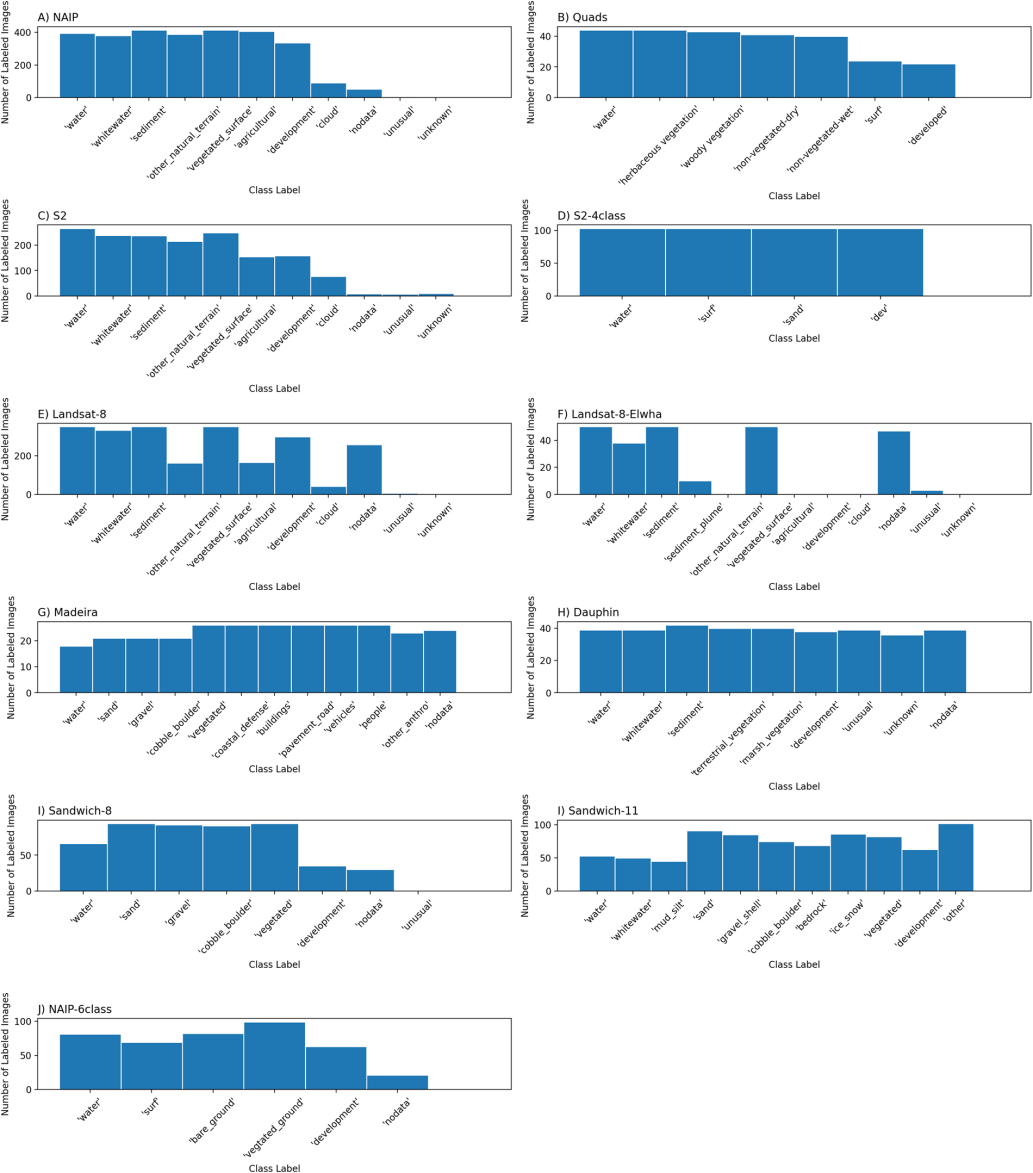
Frequency distribution of images labeled by class label.

The frequency distributions of images labeled by unique labeler ID (Fig. 5), and of images labeled by class label (Fig. 6) show that most of the dataset was labeled by three individuals (ID1, 2, and 3) and that certain datasets were labeled by others (ID4 and ID5). Further, distributions of labeler IDs by images labeled and pixels labeled (Table 4) reveal that anonymous labeling (ID6) affects 1.1% of the dataset in terms of number of total pixels labeled, or 1.6% of all images.

**Table 4:** Distributions of (anonymized) labeler IDs by images labeled and pixels labeled.

Labeler ID	Number of images labeled	Millions of pixels labeled
1	415	276.178
2	497	194.595
3	826	614.167
4	40	48.917
5	44	44.072
6 (labeler did not identify themselves)	30	14.637

**Fig. 7:**
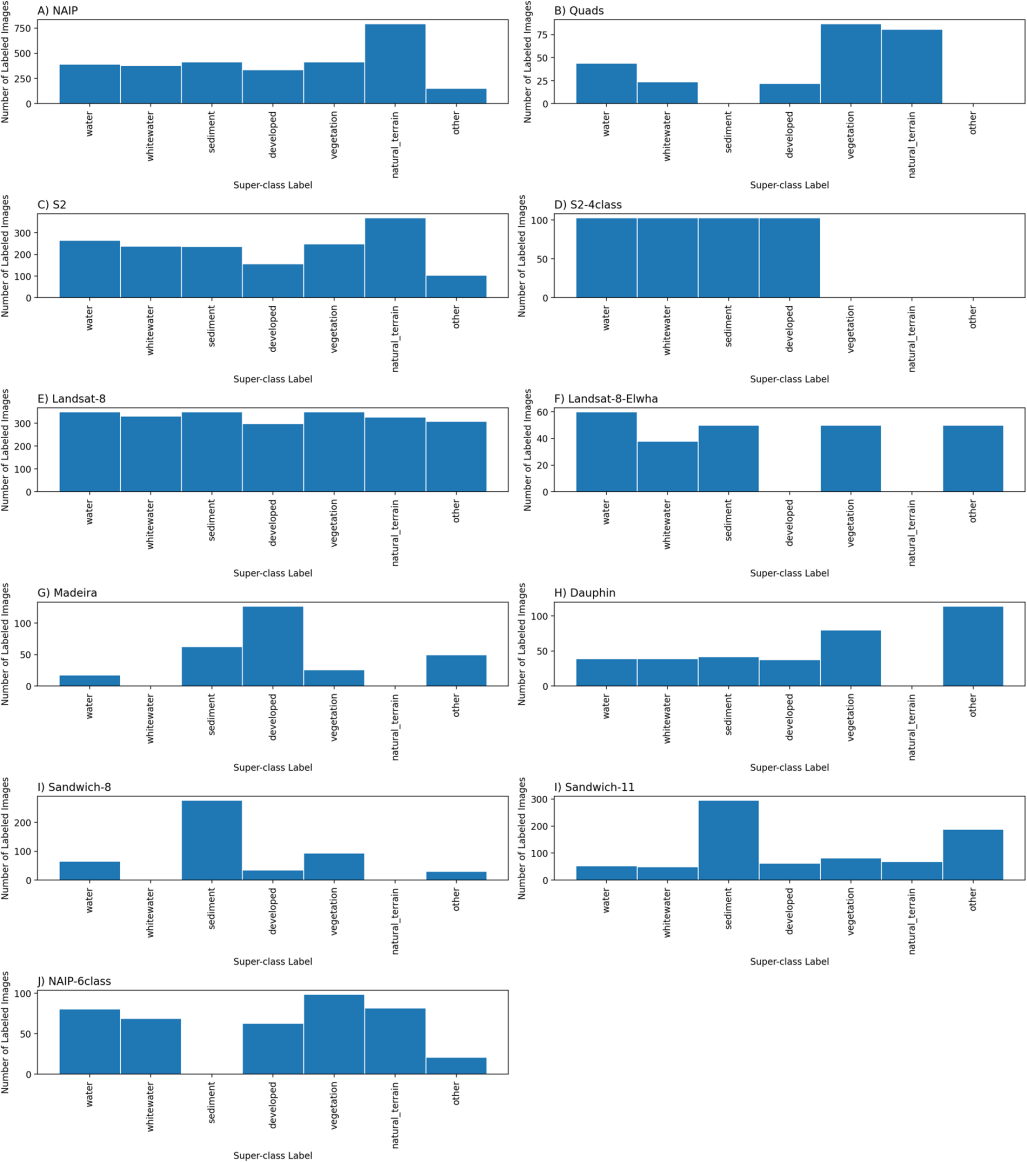
Frequency distribution of images labeled by superclass label. We define a superclass as a broad class name for a collection of component classes. There are seven superclass labels, and between four and 12 class labels depending on the dataset. Hence the empty bars in some of the frequency histograms shown. Computer codes are provided that generate superclass label image sets.

**Fig. 8:**
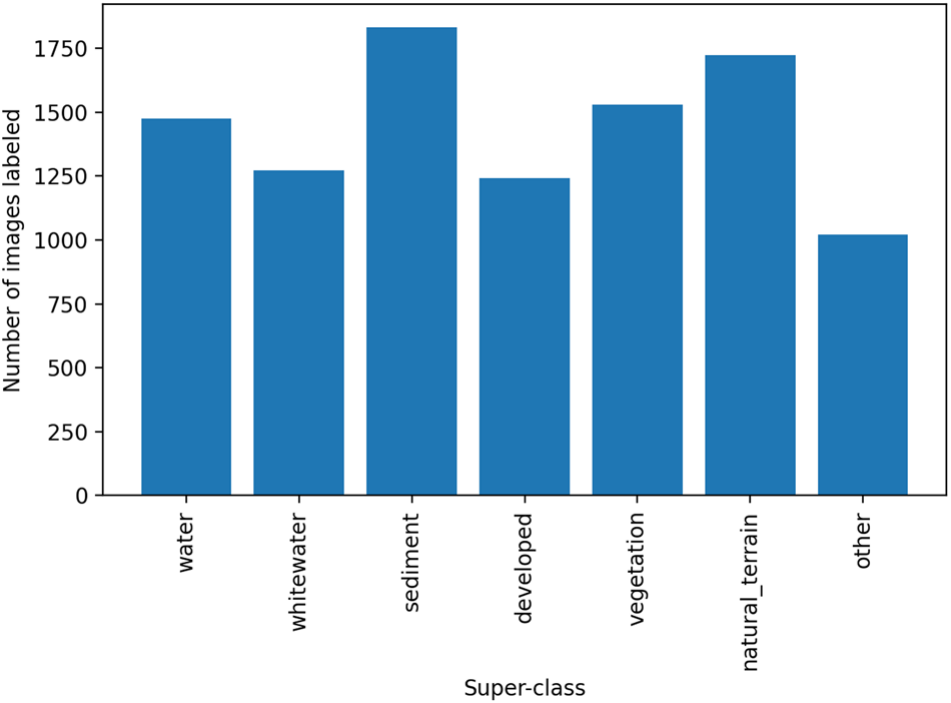
Frequency distribution of all images labeled by superclass label.

Each data record has a unique set of classes; however, labels are easily re-processed to map multiple classes to a standardized set of “superclasses” across all data records. Superclasses are broad class names for a collection of component class labels. For example, ‘buildings’ and ‘vehicles’ are a subset of the ‘developed’ superclass, and ‘sand’ and ‘gravel’ are part of the ‘sediment’ superclass. We defined seven superclass labels, and between four and 12 class labels depending on the dataset. Table 5 documents our mapping from per-set classes to superclasses. The per-set frequency distributions of labeled images by superclass label vary considerably (Fig. 7); however, the summed frequency distributions of all labeled images by superclass label are somewhat even, with all seven superclasses represented by between ~1000 and ~1800 images (Fig. 8).

**Table 5:** A mapping (look-up dictionary) between seven superclasses and the component classes used across all 10 data records.

Superclass names	Aliases (component class names)
water	water, sediment plume
whitewater	whitewater, surf
sediment	sediment, sand, gravel, gravel/shell, cobble/boulder/ mud/silt
developed	developed, dev, coastal defense, pavement/road, other anthro, vehicles, buildings, development
natural terrain	bedrock, bare ground, other natural terrain, other bare natural terrain
vegetation	vegetated, vegetated surface, vegetated ground, terrestrial vegetation, marsh vegetation, herbaceous veg, herbaceous vegetation, wood vegetation, woody veg
other	other, unknown, unusual, nodata, people, ice/snow, cloud

**Fig. 9:**
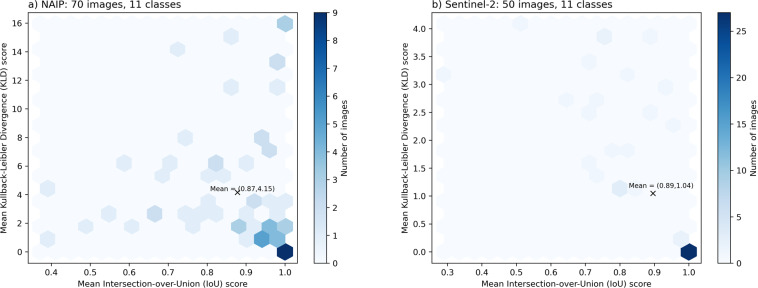
Frequency distribution of all images labeled by mean IoU and Kullback-Leibler divergence scores, for the (**a**) NAIP-11 class and (**b**) Sentinel-2 11-class datasets.

We use the methods described by a companion paper^44^ to compute mean Intersection over Union (IoU) scores for quantifying inter-labeler agreement. We use 120 images across two datasets, namely NAIP (70 image pairs) and Sentinel-2 (50 image pairs), that have been labeled independently by our most experienced labelers (Table 4), namely ID2 and ID3. Mean IoU is the standard way to report agreement between two realizations of the same label image. IoU ranges from zero to one; one indicates perfect agreement. Further, because IoU quantifies spatial overlap and is prone to class imbalance^44^, we also computed Kullback-Leibler divergence scores^47^ that quantifies agreement between class-frequency distributions. Kullback-Leibler divergence ranges from zero to one; zero indicates perfect agreement. As shown by a companion paper^44^, it is preferable to examine agreement using multiple independent metrics. Bivariate frequency distributions of all images labeled by mean IoU and Kullback-Leibler divergence scores were computed for the (a) NAIP-11 class and (b) Sentinel-2 11-class datasets (Fig. 9). The great majority of labeled images have high IoU and correspondingly low KLD scores; however, there is variability in this trend, especially for the NAIP images (Fig. 9a) because the two metrics quantify different aspects of agreement. The mean of mean IoU scores is 0.88, which is considered good agreement^44^. We recommend using 1 minus 0.88, or 0.12, as an expected irreducible error rate. Based on the finding of a companion paper^44^ that mean IoU scores tend to be inversely correlated with number of classes, we would suggest that this error is a conservative estimate. While we only present agreement statistics for only the satellite and NAIP imagery here, the interested reader is referred to that paper^44^ for identical agreement metrics on similar orthomosaic data, which makes up the bulk of the rest of the Coast Train datasets.

## Usage Notes

Below we organize additional information for users of these data records, organized by six themes. The first is the specific information need met by the data records, outlining four ways in which the data may be used for model training, added to by others, and how the label data may have inherent value in analysis of how and why humans make labeling decisions. How these data meet standards of reproducibility are discussed, before advice is given over the use of the data for image segmentation model training. Finally, we briefly review existing datasets and modeling workflows that are complementary to the present data.

### Information need

We define the information need met by Coast Train as:

1. Pixel-level discrete classification of a variety of publicly available geospatial imagery that are commonly used for coastal and other Earth surface processes research.
2. Statistics describing agreement that might be used to define uncertainty in labeled data. This uncertainty could be interpreted as the irreducible error (cf.^33^).
3. A fully reproducible workflow, facilitating end-user-defined accuracy assessments and quality control procedures.
4. An extensible and open dataset, that might be actively contributed to by others.

### Reproducibility

The outcome of this effort is a dataset useful for custom spatio-temporal classification of coastal environments from geospatial imagery using a variety of potential image segmentation methods, including a multi-purpose family of fully convolutional deep learning models, using the software Segmentation Gym (https://github.com/Doodleverse/segmentation_gym), described in another paper^48^. The present paper highlights the dataset, documents methods used to create it, and quantifies uncertainty associated with multiple labelers. Mindful of the problems that have been identified in the construction of human-labeled datasets^34^, of which possibly most alarming was the evidence that two-thirds of publications with new datasets provided insufficient detail about how their data were constructed, we have endeavoured to provide a thorough description of the process by which the dataset was constructed, including the choices and compromises made.

### Image segmentation model training

A significant advantage of Coast Train is the ability to efficiently remap classes and re-train a model without having to re-label imagery. The utility scripts contained within the Doodler program^44^ provide several means of organizing existing data but also include an approach to re-train a model with new or updated classes using previous labels. It is also possible to aggregate classes, depending on the application. For example, if a binary land-water mask is required for some application, it is possible to aggregate all land cover classes associated with land into one class representing land and all water classes aggregated into a single water class. This binary land-water classification scheme would be valuable when attempting to automate, for example, shoreline detection.

**Fig. 10:**
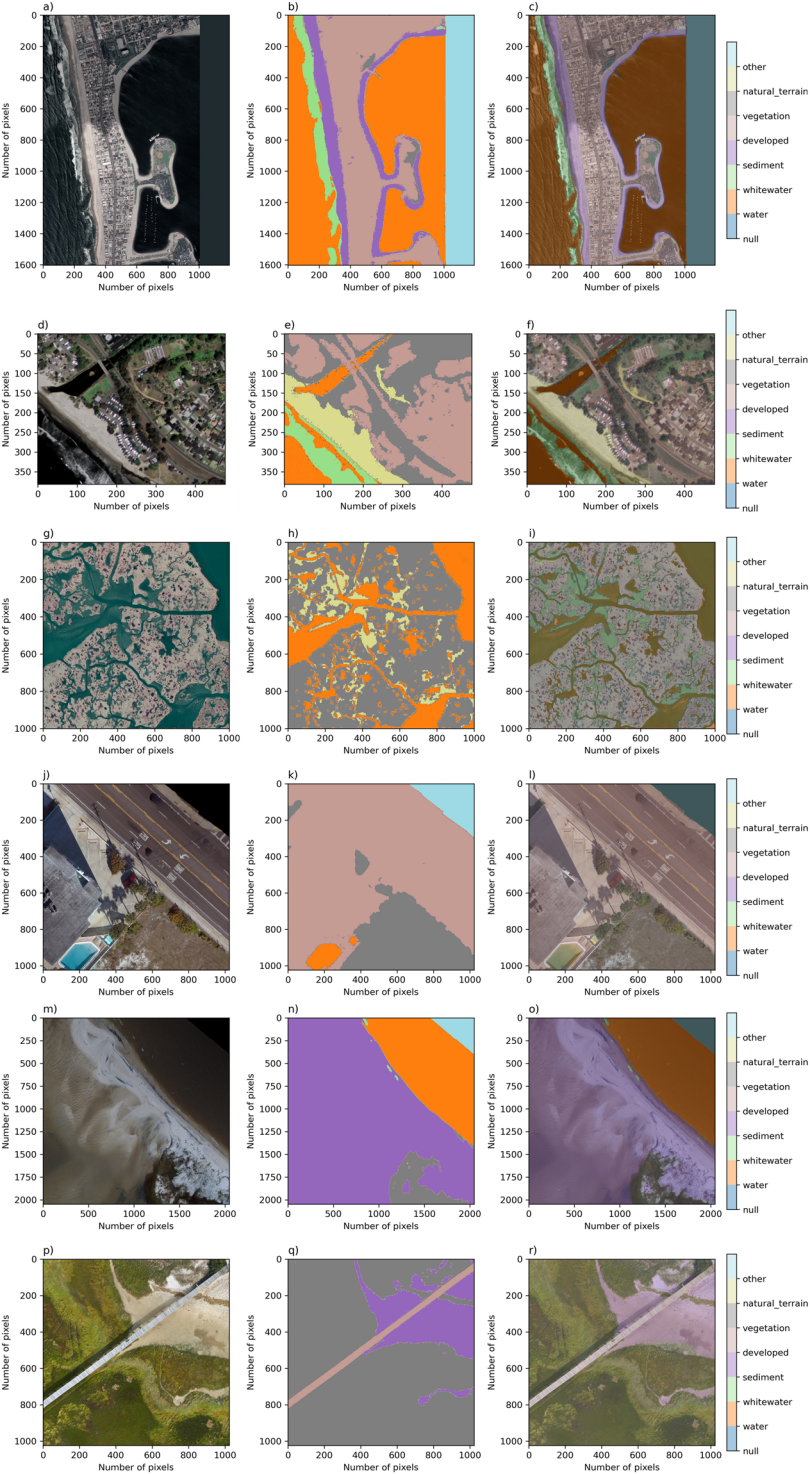
Rows (from left to right) depict one example image, corresponding label image remapped into a standardized set of classes, and image-label overlay, of each of the orthomosaic datasets. Columns show imagery from San Diego, California (**a**), Monterey Bay, California (**d**), Mississippi River Delta, Louisiana (**g**), Madeira Beach, Florida (**j**), Pelican Island, Alabama (**m**), and Sandwich Town Beach, Massachusetts (**p**).

**Fig. 11:**
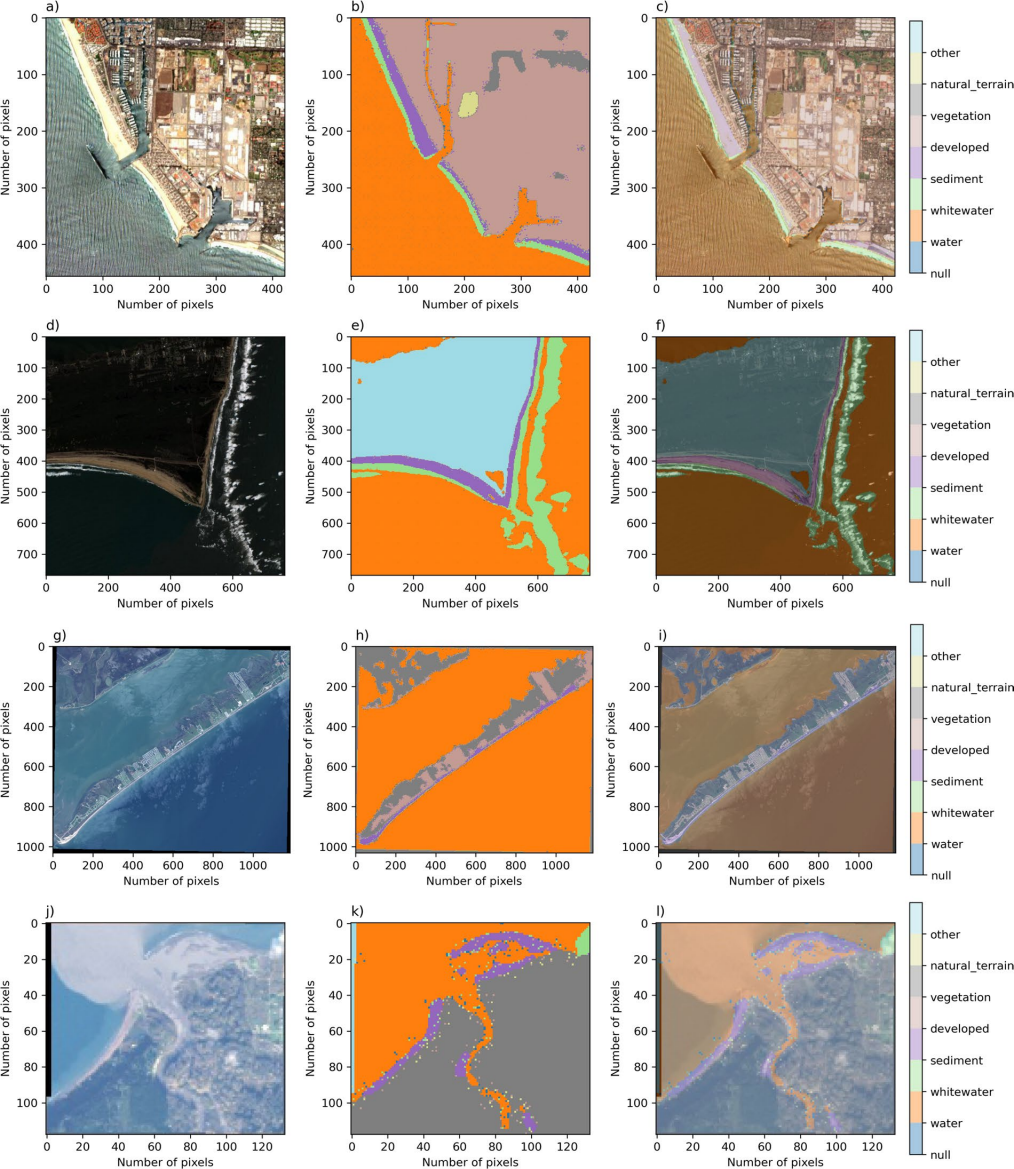
Rows (from left to right) depict one example image, corresponding label image remapped into a standardized set of classes, and image-label overlay, of each of the satellite image datasets. From top to bottom; Sentinel 2; Sentinel 2, 4 class; Landsat-8; and Landsat-8, Elwha. Columns show imagery from Ventura, California (**a**), Cape Hatteras, North Carolina (**d**), Galveston Island, Texas (**g**), Elwha River Delta, Washington (**j**).

Example superclass label images are shown for orthomosaic (Fig. 10) and satellite (Fig. 11) datasets using the mapping shown in Table 5. These may be compared to the equivalent original label images in Figs. 1, 2, respectively. Computer codes are provided (https://github.com/CoastTrain/CoastTrainMetaPlots) that generate these superclass label image sets for all images in each of the ten data records. Original classes and superclasses may have different uses, for example the use of superclass label imagery would be a ready means to train a supervised image segmentation model with broad classes on the full dataset consisting of all ten records. Individual class sets tend to contain more classes and may be more useful for image segmentation model training for more specific classes on particular image sets. In another paper^48^, we used merged class sets such as these to demonstrate and compare image segmentation model training strategies and outcomes.

### Complementary image analysis and ML tools

The data are contained in the numpy^46^ compressed data format, which is purposefully compatible with Doodler^44^, the accompanying dataset^45^, and image segmentation modeling suite, “Segmentation Gym”^48,49^. Together, Doodler, Segmentation Gym, and models created by Segmentation Gym using Coast Train data, represent a small ecosystem of compatible software tools for custom label image creation, image segmentation model application and custom training and retraining for coastal, estuarine, and wetland environments. In addition, the number and availability of open-source image processing and machine learning-based image analysis and classification methodologies specifically for coastal and estuarine environments is on the rise. For example, specially designed software packages that allow for custom mapping of coastal environments by exposing an API for custom machine learning-based mapping^26,32^.

### Complementary datasets

Coastal science has benefited from sharing of datasets^50–52^ and applications (e.g.^53,54^) have also made extensive use of national-scale LULC datasets built by governmental agencies using large satellite collections such as NOAA’s Coastal Change Analysis Program (www.coast.noaa.gov/htdata/raster1/landcover/bulkdownload/30m_lc.) and the Multi-Resolution Land Characteristics consortium (https://www.mrlc.gov/) in the United States, and a plethora of others for both general and specific needs^55,56^. These products usually result from heavily post-processed mosaics from imagery collected at multiple times, and often take several years to develop, therefore they are not always suitable for event-scale processes, observations at custom frequencies or specific times, or customized categories, all of which are so crucial in process-based studies of coasts^12^. That said, many of the aforementioned datasets could be used effectively in many contexts, including so-called “transfer learning,” where a ML model is enhanced by pre-training on one dataset then transferred to the same or similar model architecture trained on a second dataset. Of particular relevance and closest in comparison with Coast Train are labeled datasets of flooded landscapes^57–59^. Finally, while the present manuscript was in peer-review, another describing a dataset with a similar scope and name, “coastTrain”, has been published^60^. That dataset is more global in coverage. It is, however, comprised only of satellite data and its classes are more specific ecosystem types than the broader physiographic classes of the present dataset, “Coast Train”. They are therefore highly complimentary datasets, and within the scope of the intended applications of both datasets, it is possible that they may be combined to train unified models, or any models trained on each respective dataset could conceivably be used in conjunction for numerous automated mapping tasks in the coastal zone.

### Sustainability and extension

Although not nearly exhaustive or definitive, the images, doodles, and labels included in this dataset have potential application across a wide range of geographies, including but not limited to sandy coasts; rocky cliffs and platforms; wetlands, marshes, and mangroves; gravel and cobble beaches; and developed coasts (Fig. 3). The classes included in this image label library are diverse in geography and coastal environment. Future versions of Coast Train could include images from new sensors and platforms, new classes, and geographies. For example, oblique imagery from aerial platforms, and representation from very high latitude and tropical regions that each present their own particular image segmentation problems due to, for example, ice or clouds. Additionally, while our data are aimed toward segmentation tasks, they could be re-purposed for object detection or other image classification tasks.

While we acknowledge that the dataset is not global in geographic distribution, the distribution of sites and sensors within the labeled datasets presented here are potentially relevant and useful to global studies aimed at classifying the coastal zone, even if they are limited to specific coastal environments of the USA. For example, many of the classes are broad, such as water, surf, sand, vegetation, etc, which has been a successful strategy adopted by many well-known and well-cited satellite image segmentation approaches in the coastal zone, such as those behind CoastSat^26^, and those used by Luijendijk *et al*.^25^, which we note were trained on significantly less imagery than is contained in the Coast Train dataset, yet still applied successfully to multiple countries, regions, and in the case of the Luijendijk *et al*.^25^ study, the entire world. As-yet unspecified Machine Learning models based on these data may or may not generalize to the entire world. However, in numerous specific situations and locations, the distribution of our labeled imagery may not transfer well to all global environments, and indeed coarse, muddy, and coral coasts are absent, as we note above.

## Data Availability

All the figures presented in this manuscript may be generated using computational notebooks provided (https://github.com/CoastTrain/CoastTrainMetaPlots). Utilities for npz file variable extraction and class remapping are provided in the Doodler^44^ and Segmentation Gym^48^ software packages. All labels were created with Doodler^44^. Imagery was downloaded using CoastSat (https://github.com/kvos/CoastSat) and Geemap (https://github.com/giswqs/geemap) functionality. For more information, please see the Coast Train project website (https://coasttrain.github.io/CoastTrain/).
